# Barriers and Facilitators to Peer Support Services for Patients With Opioid Use Disorder in the Emergency Department

**DOI:** 10.7759/cureus.23145

**Published:** 2022-03-14

**Authors:** Emily Kauffman, Yuxuan Qiu, Jennifer A Frey, Jason J Bischof

**Affiliations:** 1 Emergency Medicine, The Ohio State University Wexner Medical Center, Columbus, USA

**Keywords:** substance use disorder, substance-related disorders, peer support, peer mentor, narcotic dependency, opioid epidemic, peer recovery coaches

## Abstract

There is a high prevalence of opioid use disorder in the United States, and emergency departments (EDs) play multiple vital roles in providing care to help these patients with achieving sobriety, one of which is the application of peer recovery services. This technical report discusses the utilization of peer recovery supporters in the ED and associated barriers. They include but are not limited to the difficult hiring process, referral process challenges for certain populations, difficulty with follow-up data collection, retention of peer recovery supporters, and a lack of ED provider awareness. This article also discussed strategies to address these barriers. Examples include simplifying hospital onboarding processes for peer recovery supporters, obtaining grants to utilize peer recovery services, and using managed care organizations to facilitate follow-up data collection, ED provider education, and discharge planning.

## Introduction

There is a high prevalence of opioid use disorder (OUD) and a high rate of prescription opioid misuse in the United States [1]. Patients often present to the emergency department (ED) with complaints such as opioid withdrawal, acute intoxication, medical complications related to OUD, or unrelated concerns. ED providers have the opportunity to initiate appropriate treatment and assist these patients with resources for OUD and to prevent medical and social complications associated with OUD. There have been many policies addressing the opioid crisis on the federal level [2]. Nationwide, ED providers have started to change their clinical practice to address OUD, including increased screening, use of brief interventions, and obtaining training in ED-initiated treatment of OUD with buprenorphine [3,4]. One of the practices gaining momentum is peer recovery services (or peer support services), which pair patients struggling with substance use disorder with individuals who have lived experience with addiction, to reduce opioid-related harms such as preventing overdose and relapse [5,6]. Some hospitals have initiated peer recovery services in their EDs to engage the high-risk population of patients with OUD [7].

Limited studies have demonstrated that the peer support process is as effective as clinical treatment and it decreases the need for psychiatric hospitalization and length of hospitalization if required [8-10]. Despite the potential benefits of peer recovery services, substantial barriers exist. This report addresses many of these barriers: the hospital onboarding process of peer recovery service staff, patient identification and referrals, patient outcomes, and barriers in the service establishment and utilization. Additionally, strategies to mitigate the aforementioned barriers will be discussed.

## Technical report

Peer recovery supporter certification, hiring, and barriers with hospital onboarding

Peer recovery services have a broader role than simply engaging with patients with OUD; they are individuals who provide shared experience and support to patients dealing with any number of substance use disorders or mental health disorders [11]. Services are varied including peer support, encouragement of self-determination and personal responsibility, support for health and wellness, addressing hopelessness and stigma, communication with providers, illness management, and friendship and leisure activities [12]. The core workforce within peer recovery services are the peers, also referred to as peer supporters or peer recovery supporters. It is a somewhat lengthy process to be certified and hired as a peer supporter. The primary requirement for the peers is to be in recovery from substance use and/or mental health disorders. Beyond the need for recovery, there are multiple pathways to becoming a peer recovery supporter. In Ohio, the certification process requires an individual to have at least three years of working or volunteering experience in a similar peer supporter role or to complete the 40-hour training that includes topics such as ethics, human trafficking, and trauma-informed care in addition to completing a 16-hour course, passing the Ohio Mental Health and Addiction Services (OMHAS) Peer Recovery Services exam, signing a code of ethics, and completing a Bureau of Criminal Investigations (BCI) background check [13]. There are multiple disqualifying offenses that prevent certification including aggravated murder, child abuse, human trafficking, and rape, amongst others. If the applicants do not have qualifying experience, this training may be prohibitive due to the time or financial resources needed to complete these tasks (i.e., lack of income during the training period). Notably, certification and training requirements vary by state.

Although the certification process appears straightforward, there are still many caveats to the hiring and onboarding processes at the employing agency level, which pose potential barriers for implementing peer recovery services in many EDs. After becoming a certified peer recovery supporter, the process of getting onboarded by a hospital can be complex. Administrative requirements will vary based on the model of staffing: direct hires versus contract hires. Organizations that hire peers implement background checks prior to hiring. Additionally, some hospitals that are contracting peer recovery services require a separate, institution-specific background check to be performed for consultants and vendors, which may further delay employment. Another factor for consideration is that many applicants have a criminal background, which is often linked to their prior substance use. Some hospitals encounter difficulty hiring peers due to criminal backgrounds related to their prior substance use or mental health issues, even though the individuals are cleared by the certification process and the hiring peer recovery service organization. In one case, a hospital navigated this barrier by implementing a more streamlined process to expedite onboarding, which mainly is to entrust the peer recovery service organization’s background check and provide explanations of any criminal background to the hospital.

One proposed solution is to streamline the background check process so that the contracted peer recovery services agency and the employing hospital use the same background check, as this will minimize duplicative efforts and reduce delays in hiring.

Patient referral process and barriers in engaging patients with peer recovery service

There are multiple pathways of referral to peer recovery services for patients with substance use disorders. This can be done via social workers, ED providers, police, and self-referral by patients. Patients can be referred directly from their ED or observation visit with linkage to peer services prior to their discharge, if appropriate. Patients may also be referred from inpatient or outpatient settings.

The operation of peer recovery services varies among EDs. For example, the demand and resources may justify the 24/7 staffing of peers in emergency departments hit especially hard by the opioid crisis. In other departments, it may be most appropriate to staff the ED with peers overnight and have the ED social workers manage the patients during the day. In a mixed approach model, peer supporters are not available 24/7, but other community support services, which consist of mental health nurses and outreach coordinators, are available when the peers are not present. A final peer recovery services model involves staffing peer supporters as an “on-call” service with the expectation that a peer arrives at the ED within 30 minutes of consultation. Our community ED currently employs three peer supporters that provide in-person coverage seven days per week from approximately 7 am to 3 am.

This process is certainly not without other barriers for patients. Patients may need to be insured or eligible for Medicaid to utilize peer recovery services in the community after discharge. This may represent a significant challenge in providing these services to patients that are the most in need as there are only 11 states in which peer recovery services may be billed to Medicaid [14]. Lack of insurance may be overcome by funding the peer recovery services through alternate means such as grants or hospital funds. Therefore, it is difficult for hospitals and other agencies to offer peer recovery services unless they are set up to bill Medicaid, support the programs with other funds, or utilize volunteers.

It is difficult to determine in the ED whether a patient is eligible for Medicaid due to the variability of Medicaid eligibility across states, and it is beyond the scope of this article to discuss ways to ensure Medicaid eligibility for patients. The solution may require hospitals to obtain multi-faceted support, including federal, state, or private funding, or for the hospital to offset the cost themselves. Examples include the Substance Abuse and Treatment Block grant (SAPT), the Access to Recovery Program (ATR) Wisconsin State Assembly Bill 455, which established a respite center for patients with mental illness or substance use disorders, and Oklahoma Senate Bill 713, which mandated their Department of Mental Health and Substance Abuse to establish a drop-in center with peer support for veterans [14,15]. Substance Abuse and Mental Health Services Administration (SAMHSA) has also made recommendations regarding funding peer recovery services, including educating state and treatment providers regarding available funds, making regulatory changes to allow the state greater flexibility to provide funding, and increasing the funds throughout the care continuum [15]. Additionally, hospitals should partner with community-based peer support agencies to improve the coordination of care for addiction upon hospital discharge.

Patient’s follow-up rate with office-based opioid treatment and systematic barrier in outcome data obtainment

While reporting the outcome data regarding rates of follow-up with office-based opioid treatment (OBOT) on patients who utilized peer recovery services would be ideal, it is often very challenging to obtain as many individuals engage and disengage with multiple treatment providers over the varying length of time. Also, these same individuals often lack housing, reliable phones, transportation, and maybe incarcerated due to their underlying addiction. Discussion of these barriers may help develop streamlined processes or serve as guidance for future studies.

One of the biggest barriers is the logistics of information sharing. Treatment centers use a variety of electronic medical record systems, which typically do not interface with hospital systems. Furthermore, 42 CFR (Code of Federal Regulations) Part 2 mandates confidentiality of records for patients with substance use disorders and allows to impose fines for any violations [16]. Due to these regulatory restrictions, information sharing between hospitals, peer services, and treatment centers is limited. These restrictions on information sharing make it very difficult to coordinate care for providers in outpatient clinics or peer recovery services and lead to providers relying on treatment history from the patient alone, which can be unreliable or incomplete. Contracted peer support agencies may not have access to treatment records or electronic medical records and thus are required to obtain a Release of Information for all patients accepting peer services in the ED.

Regarding solutions for this, there are companies (e.g. Cordata and Ascend) developing platforms that will facilitate sharing patient data from treatment centers. It will also aid peer recovery service programs as they work with managed care organizations to determine if the patient is currently engaged in outpatient treatment as well as tracking patient follow-up.

Barriers with peer retention, provider awareness, and patient disposition

Staff retention is a challenge for peer recovery services. Although it is a rewarding career, peers may find it difficult to work in the ED due to irregular shifts (such as overnights), increased stress related to repeat exposure to patients in crisis, and visual cues such as syringes for those with prior history of intravenous drug use [17]. The ED may also trigger previous mental health issues, coined as “caregiver trauma” for some peers causing them to leave the ED work setting. Moreover, delayed hiring due to additional background checks also poses an obstacle for peers who may struggle to find employment and maintain a basic cost of living. Finally, some peer supporters may leave due to higher-paying jobs as they obtain experience and additional education or training.

Another challenge for the implementation of peer recovery services in the ED is provider awareness. While many ED providers in hospitals that utilize peer service are familiar with their existence, some hospital staff still have difficulty with role definitions. Most importantly, peer recovery supporters are not licensed social workers, as they are not trained to provide resources for home health, transportation, etc. This gap in understanding can be overcome by educating ED providers about the peer supporter roles or providing orientation materials. Education of healthcare professionals on the role of peer support is especially important if considering expanding their services to the inpatient hospital setting where integration is key for multidisciplinary care (social work, case management, bedside nursing) for these complex patients.

As many EDs track their disposition time, unrealistic expectations may be placed on peers to facilitate a quick disposition. Discharge plans may be complex for patients with substance use disorders. Although the patient may not have a life-threatening intoxication or withdrawal syndrome, it does not necessarily mean that it is safe to discharge the patient back home or to the street. Utilization of peer recovery services is helpful to discuss options with patients; however, due to the lack of availability of inpatient treatment centers or outpatient clinic appointments, the disposition can be lengthy as peer supporters assist ED providers to find linkage of services for the patient. A similar approach is often taken regarding patients presenting to the ED for mental health crises, where dispositions are often delayed due to the limited availability of inpatient psychiatric beds. One solution may be to first increase the disposition of patients to clinical decision units or observation units so peers may have more time to facilitate linkage and the final disposition. This solution will also mitigate the risk of harm by discharging these patients after an unintentional overdose or other high-risk scenarios [18].

## Discussion

The opioid epidemic continues to devastate the United States, requiring innovative and multi-faceted treatment models. Peer recovery services are an integral component of treatment and recovery that requires additional focus. Multiple barriers remain to the widespread implementation of such services in the hospital setting [18,19]. Although many barriers are likely ubiquitous (hiring of qualified peers), state regulations and variable practice settings such as the ED provide unique challenges.

Due to the complexity of the epidemic, solutions often require tailoring to the needs of local settings and the patient. As noted in this technical report, multiple approaches can be employed to engage patients resulting in several unique barriers related to the initiation of services and subsequent coordination of care upon disposition of ED patients. One study examined median response time across 20 hospitals from initial referral to peer supporter arrival at the bedside to be eight minutes, which may be largely dependent on the operational and staffing model used [20].

Peers serve an essential role in the care of patients with substance use disorder and should be recognized as such by the clinical care team. In addition to care coordination barriers, the chaotic environment and high staff turnover rates in the ED setting may provide a distinct obstacle to establishing a structured peer recovery supporter program. As such, education of the providers in addition to patients regarding the role of peer recovery services is essential for a successful implementation.

The peer supporter role is notable in patient care settings, as their primary purpose is to offer and be open about their lived experience, while most healthcare professionals are taught to maintain professional boundaries [18]. Establishing boundaries with patients can be a challenge for peers and those who supervise them. Thus, it is important that colleagues and services agencies treat peer supporters as equal members of the team while recognizing their individual knowledge and experiences. While often history of addiction and involvement with criminal justice is looked down upon, these experiences are exactly what enables peer recovery supporters to make a positive impact in patients’ lives.

These many challenges and barriers require further investigation with an emphasis on clinical outcomes and further investment as noted in this technical report [19]. Despite these limitations, data from inpatient peer recovery services have demonstrated improved clinical outcomes through a reduction in hospital readmissions and ED visits [20]. Other studies have focused on the impact of peer services on substance use, such as toxicology reports or treatment retention [21]. However, there is also a need to assess the impact of peer services on measures of daily functioning, self-efficacy, and quality of life, as we aim to implement a harm-reduction approach to addiction treatment. It is crucial that peer recovery supporters and their employing agencies support multiple pathways to recovery and recognize addiction as a chronic disease.

## Conclusions

As the opioid epidemic continues to ravage communities, the advent of peer recovery services is important to encourage patients in recovery, engage patients in supportive services, and link patients with substance use disorders to appropriate inpatient and outpatient care. The barriers of applying peer recovery services into ED practice include difficulty with hiring peers, referral process, especially for non-insured and non-Medicaid patients, difficulty of obtaining follow-up data due to current federal regulations surrounding information sharing, peer retention, provider awareness, and sometimes unrealistic expectations of ED door-to-disposition time. Proposed solutions include simplifying the hospital onboarding process by merging background checks, obtaining grants or having hospitals offset costs, using managed care organizations, providing education to ED providers, and utilizing observation units and other ED areas to support safe discharge planning.
